# Supplementary material for: Unraveling the dislocation core structure at a van der Waals gap in bismuth telluride
Source: Nat Commun. 2019 Apr 23;10:1820. doi: 10.1038/s41467-019-09815-5 (PMC6478677; doi:10.1038/s41467-019-09815-5)
Supplement: Supplementary file 1 — Supplementary Information [file 41467_2019_9815_MOESM1_ESM.pdf]

Supplementary Information

Unraveling the dislocation core structure at a van der Waals gap in bismuth telluride

D. L. Medlin et al.

Supplementary Note 1: Semidiscrete variational Peierls-Nabarro model

The dislocation disregistry vector δ_i (index i labels the Cartesian components) is defined as the difference in displacement between the material just above and just below the slip plane containing the dislocation. Here, we consider a perfectly straight dislocation with the line parallel to the z-direction and the slip plane normal to the y-direction. If the dislocation is planar, i.e., only spreads along the slip plane, then δ_i is only a function of the x-coordinate. Using this coordinate system, δ_1 corresponds to the edge component of the disregistry, δ_3 to the screw component, and δ_2 to the out-of-plane component.

The classical Peierls-Nabarro model [1, 2] represents the dislocation core by a continuous spread of infinitesimal Volterra dislocations. Thus $\delta_i(x)$ is a continuous function satisfying the boundary conditions $\delta_i(\infty) - \delta_i(-\infty) = b_i$, where b_i is the dislocation Burgers vector. By assuming a particular functional form for $\delta_i(x)$, the dislocation energy can be evaluated by integrating the interactions of the infinitesimal dislocations with each other and with the crystal over all x and adding the misfit energy.

In the semidiscrete variational Peierls-Nabarro (SDVPN) model [3–5], the disregistry δ_i is only evaluated at discrete values of x that are equally spaced by Δx. Typically, Δx is taken to correspond to a spacing of atomic columns along the x-direction, giving the resulting model an atomic character. The full dislocation is represented by a number of equally spaced partial dislocations. Their density $\rho_i(x)$ is defined as the numerical derivative of the disregistry,

$$\rho_i(x) = \frac{\delta_i(x) - \delta_i(x - \Delta x)}{\Delta x}, \tag{1}$$

which is assumed to be constant between the evaluated x coordinates. By its nature and definition,

$$\sum_x \rho_i(x)\Delta x = b_i. \tag{2}$$

The total energy of the SDVPN dislocation at zero applied stress is represented as the sum of the energy arising from the dislocation's elastic strain field and the crystal misfit due to the dislocation spreading:

$$E_{\text{total}} = E_{\text{elastic}} + E_{\text{misfit}}. \tag{3}$$

Both terms are expressed as functions of $\delta_i(x)$, which allows for the disregistry profile to be solved for by minimizing the total energy.

The elastic energy of the dislocation accounts for the interaction energy between all α-β pairs of partial dislocations positioned at the N evaluated x-coordinates:

$$E_{\text{elastic}} = \frac{1}{4\pi} \sum_{\alpha=1}^{N} \sum_{\beta=1}^{N} \chi(\alpha, \beta)\, K_{ij} \rho_i(x^\alpha)\, \rho_j\left(x^\beta\right), \tag{4}$$

where K_{ij} is the energy coefficient tensor and χ is a multiplicative factor that depends on the distance between the dislocation positions x^α and x^β:

$$\chi(\alpha,\beta) = \frac{3}{2}(\Delta x)^2 + \psi(\alpha-1,\beta-1) + \psi(\alpha,\beta) - \psi(\alpha,\beta-1) - \psi(\beta,\alpha-1), \quad (5)$$

where

$$\psi(\alpha,\beta) = \frac{1}{2}(\alpha-\beta)^2(\Delta x)^2 \ln(|\alpha-\beta|\Delta x). \quad (6)$$

The leading coefficient in Eq. (4) depends on how K_{ij} is defined. Here, K_{ij} is taken such that in the isotropic elasticity case $K_{11} = K_{22} = \mu/(1-\nu)$ and $K_{33} = \mu$, where μ and ν are the shear modulus and Poisson's ratio, respectively.

The misfit energy is obtained by mapping the disregistry profile onto the gamma-surface (i.e., the generalized stacking fault energy γ as a function of the translation vector):

$$E_{\text{misfit}} = \sum_{\alpha=1}^{N} \gamma[\delta_i(x^\alpha)]\,\Delta x. \quad (7)$$

The γ function can either be 2D, in which case it only depends on the in-plane components of the disregistry, δ_1 and δ_3, or 3D, in which case it includes the out-of-plane disregistry component δ_2. Some SDVPN models include an additional correction term to account for either the surface effect of the slip plane [6, 7] or non-local interactions between the evaluated positions [8, 9]. Neither correction was included here as they require assumptions of the dislocation symmetry along the slip plane or are numerically fitted using atomistic data.

The anisotropic energy coefficient tensor K_{ij} was obtained by solving the Eshelby elastic model for a straight dislocation [10] using the Stroh method [2, 11, 12] with elastic constants calculated with each of the DFT functionals. The resulting K_{ij} has the form

$$K_{ij} = \begin{bmatrix} K_{11} & K_{12} & 0 \\ K_{12} & K_{22} & 0 \\ 0 & 0 & K_{33} \end{bmatrix}. \quad (8)$$

The four non-zero elements represent a screw component K_{33}, an edge component in the slip plane K_{11}, an edge component perpendicular to the slip plane K_{22}, and a edge coupling component K_{12}. For all sets of the elastic constants C_{ij} explored here, the general trends in the K_{ij} components are that K_{11} and K_{22} are comparable but not equal, K_{33} is slightly smaller than the edge components, and K_{12} is roughly an order of magnitude smaller than the other components.

Solving for the dislocation disregistry requires inputs for $\gamma(\delta_i)$ and K_{ij} at the relaxed lattice parameter a. These values were obtained using four different exchange-correlation

functionals: LDA [13], optPBE-vdW and optB88-vdW [14–17], and DFT-D2 [18]. Table 1 summarizes the lattice and elastic constants of Bi_2Te_3 computed by DFT in comparison with experiment. (Note that for rhombohedral crystals such as Bi_2Te_3, $2C_{66} = C_{11} - C_{12}$.) While the lattice constant displays decent agreement across the DFT functionals, the elastic constants show more variability, including C_{13} and C_{33}. The sensitivity of C_{13} and C_{33} to the choice of the DFT functional is due to the component 3 direction being normal to the van der Waals-bonded layers in the Bi_2Te_3 crystal structure.

An initial disregistry guess was made using an arctangent expression similar to the one used in the classic Peierls-Nabarro model,

$$\delta_i(x) = S\frac{b_i}{\pi}\arctan\frac{x}{\xi} + \frac{b_i}{2}, \tag{9}$$

where ξ is the dislocation halfwidth and S is a scaling factor introduced to satisfy the boundary conditions

$$\delta_i(x(0)) = 0, \;\; \delta_i(x(N)) = b_i \tag{10}$$

despite the fact that $x(0)$ and $x(N)$ are finite. The ξ value used in the initial disregistry was the one that minimized Eq. (3) using the disregistry of the form of Eq. (9).

For the calculations performed here, we chose $N = 300$ and $\Delta x = a\sqrt{3}/3$, which resulted in x values spanning the range ± 38 nm. This wide range was necessary to ensure that the boundary conditions did not affect the dislocation core with its large spreading. As the gamma-surface obtained by the DFT calculations only considered the in-plane translations, the out-of-plane disregistry component δ_2 was excluded from the calculations. As a result, the energy coefficient tensor only included the K_{11} and K_{33} components. Starting with the initial guess (9), the disregistry profile was optimized using Powell's minimization method [23] subject to the boundary conditions (10). For each calculation, the minimization algorithm was restarted ten times for better convergence.

Supplementary Note 2: Burgers circuit construction

The Burgers vectors of the experimentally observed dislocations were determined by circuit mapping. This approach consists of constructing a closed path of crystallographic translation vectors around the atomically resolved image of the defect. Using the FSRH convention [2], with the line direction out of the board, the Burgers vector $\mathbf{b}$ is given by:

$$\mathbf{b} = -\sum_i \mathbf{C}_i,$$

where $\mathbf{C}_i$ are the individual segments of the circuit path.

	LDA [13]	optPBE-vdW[14–17]	DFT-D2 [18]	optB88-vdW [14–17]	Experim. [19–22]
a (nm)	0.4357	0.4465	0.4329	0.4445	0.438
c (nm)	2.983	3.159	3.138	3.106	3.050
C_{11} (GPa)	81.9	61.3	73.1	67.1	74.36
C_{12} (GPa)	21.6	15.9	20.9	17.9	21.98
C_{13} (GPa)	32.2	16.3	28.3	21.3	29.17
C_{14} (GPa)	19.4	9.4	15.5	12.5	15.41
C_{33} (GPa)	57.9	26.5	47.1	35.4	51.60
C_{44} (GPa)	37.8	17.5	27.4	23.4	31.35
C_{66} (GPa)	30.15	22.7	26.1	24.6	26.19
γ_{SF} ($\mathrm{mJ\,m^{-2}}$)	92	29	34	46	

Supplementary Table 1 Lattice parameters (a and c), elastic constants (C_{ij}) and basal-plane stacking-fault energy (γ_{SF}) of Bi_2Te_3 predicted by DFT calculations with different exchange-correlation functionals in comparison with experimental data [19–22]. The experimental values of a and c [19–21] and C_{ij} [22] were extrapolated to 0 K. No experimental values of γ_{SF} are available.

In the example shown in Figure 1c, segments **bc** and **da** are of equal crystallographic length but opposite direction and hence cancel. On the other hand, **ab** and **cd** have different crystallographic lengths resulting in a finite Burgers vector. Specifically, $\mathbf{ab} = 21 \times (a/2)[01\bar{1}0] \pm (a/6)[2\bar{1}\bar{1}0]$ and $\mathbf{cd} = 20 \times (a/2)[0\bar{1}10]$. Here, the ambiguity of $\pm(a/6)[2\bar{1}\bar{1}0]$ is because the image is insensitive to positions along the imaging projection and hence displacements along this direction can only be inferred from crystallographic considerations. The resulting Burgers vector is hence either $\mathbf{b} = (a/3)[\bar{1}\bar{1}20]$ or $\mathbf{b} = (a/3)[1\bar{2}10]$, which in either case is a full lattice dislocation with its Burgers vector lying in the basal plane at 60° with respect to the $[2\bar{1}\bar{1}0]$ line direction.

Supplementary Note 3: Effect of the stacking fault on electronic properties.

To demonstrate the possible effect of the dislocation core structure in Bi_2Te_3 on electronic properties, DFT calculations (using the optB88-vdW exchange-correlation functional) have been performed for a Bi_2Te_3 slab containing six quintuplet layers (we employ a hexagonal unit cell with 15 atoms). We considered two systems: one without stacking faults and another with a stacking fault in the middle of the slab. The stacking fault structure is representative of the dislocation core structure. The Supplementary Figure 1(a,b) shows the electronic band structure of the two systems, presented in the form of a spectral function for an energy range near the Fermi level (set to zero) and for wave-vectors near the Γ point along the Γ-M direction of the Brillouin zone of the corresponding hexagonal reciprocal space. In the system without the stacking fault (panel a), the overall bands tructure is in agreement with that obtained in previous DFT studies (compare to Fig. 4a in [24]). We note the presence of topological surface states intersecting at the Dirac point located near zero energy at the Γ point. These states are not affected significantly by the presence of the stacking fault in the middle of the slab. To disentangle the surface states from the rest we show in Supplementary Figure 1(c-d) the electronic band structure of the two systems projected on one of the middle quintuplets. In the absence of a stacking fault (panel c) the bulk-like states show a gap at the Γ point of about 0.25 eV. These states are impacted by the stacking fault, which leads to a valence-conduction gap separation becoming significantly smaller, namely about 0.18 eV. We believe that this local band gap renormalization is due to the change in local strain caused by the stacking fault. Indeed, previous *ab initio* calculations have shown that the band gap of bulk Bi_2Te_3 at the Γ point tends to decrease when subject to uniaxial tensile strain [25]. We find that the quintuplet separation at the stacking fault is about 10% larger than in the absence of the stacking fault. This expansion can be deduced from a plot of the electron charge density distribution function, as shown in Supplementary Figure 1 for a particular isovalue surface (about a quarter of the maximum value). Comparing the two cases it is apparent that the

structural relaxation in the stacking fault region increases the gap between the quintuplet layers. The local renormalization (decrease) of the band gap is likely to impact (increase) the concentration of free charge carriers near the stacking fault. A more detailed analysis (outside the scope of this work) is needed to better understand the impact of dislocation cores on the electronic transport properties of Bi_2Te_3.

Supplementary References

[1] Nabarro, F. R. N. *Theory of Crystal Dislocations* (Dover, New York, 1987).

[2] Hirth, J. P. & Lothe, J. *Theory of Dislocations* (Wiley, New York, 1982), 2 edn.

[3] Bulatov, V. V. & Kaxiras, E. Semidiscrete variational Peierls framework for dislocation core properties. *Phys. Rev. Lett.* **78**, 4221–4224 (1997).

[4] Lu, G., Kioussis, N., Bulatov, V. V. & Kaxiras, E. Generalized-stacking-fault energy surface and dislocation properties of aluminum. *Phys. Rev.* B **62**, 3099–3108 (2000).

[5] Lu, G., Kioussis, N., Bulatov, V. V. & Kaxiras, E. The Peierls-Nabarro model revisited. *Philos. Mag. Lett.* **80**, 675–682 (2000).

[6] Wang, S. F. A unified dislocation equation from lattice statics. *J. Phys.* A*: Math. Theor.* **42**, 025208 (2009).

[7] Wang, R., Wang, S. F., Wu, X. Z. & Wei, Q. Y. First-principles determination of dislocation properties in magnesium based on the improved Peierls-Nabarro equation. *Physica Scripta* **81**, 065601 (2010).

[8] Miller, R., Phillips, R., Beltz, G. & Ortiz, M. A non-local formulation of the Peierls dislocation model. *J. Mech. Phys. Solids* **46**, 1845–1867 (1998).

[9] Liu, G. S., Cheng, X., Wang, J., Chen, K. G. & Shen, Y. Improvement of nonlocal Peierls-Nabarro models. *Comp. Mater. Sci.* **131**, 69–77 (2017).

[10] Eshelby, J. D., Read, W. T. & Shockley, W. Anisotropic elasticity with applications to dislocation theory. *Acta Metall.* **1**, 251–259 (1953).

[11] Stroh, A. N. Dislocations and cracks in anisotropic elasticity. *Philos. Mag.* **3**, 625–646 (1958).

[12] Stroh, A. N. Steady state problems in anisotropic elasticity. *J. Math. Phys.* **41**, 77–103 (1962).

[13] Kohn, W. & Sham, L. J. Self-consistent equations including exchange and correlation effects. *Phys. Rev.* **140**, A1133–A1138 (1965).

[14] Dion, M., Rydberg, H., Schroder, E., Langreth, D. C. & Lundqvist, B. I. Van der Waals density functional for general geometries. *Phys. Rev. Lett.* **92**, 246401 (2004).

[15] Klimeš, J., Bowler, D. R. & Michaelides, A. Chemical accuracy for the van der Waals density functional. *J. Phys.: Condens. Matter* **22**, 022201 (2010).

[16] Klimeš, J., Bowler, D. R. & Michaelides, A. Van der Waals density functionals applied to solids. *Phys. Rev.* B **83**, 195131 (2011).

[17] Román-Pérez, G. & Soler, J. M. Efficient implementation of a van der Waals density functional: Application to double-wall carbon nanotubes. *Phys. Rev. Lett.* **103**, 096102 (2009).

[18] Grimme, S. Semiempirical GGA-type density functional constructed with a long-range dispersion correction. *J. Comput. Chem.* **27**, 1787–1799 (2006).

[19] Adam, A. Rietveld refinement of the semiconducting system $Bi_{2-x}Fe_xTe_3$ from X-ray powder diffraction. *Materials Research Bulletin* **42**, 1986–1994 (2007).

[20] Lange, P. Ein Vergleich zwischen Bi_2Te_3 und Bi_2Te_2S. *Naturwissenschaften* **27**, 133–134 (1939).

[21] Nakajima, S. The crystal structure of $Bi_2Te_{3-x}Se_x$. *J. Phys. Chem. Solids* **24**, 479–485 (1963).

[22] Jenkins, J. O., Rayne, J. A. & Ure, R. W. Elastic moduli and phonon properties of Bi_2Te_3. *Phys. Rev.* B **5**, 3171–3184 (1972).

[23] Powell, M. J. D. Efficient method for finding minimum of function of several-variables without calculating derivatives. *Computer Journal* **7**, 155–162 (1964).

[24] Forster, T., Kruger, P. & Rohlfing, M. GW calculation for Bi_2Te_3 and Sb_2Te_3 thin fims: Electronic and topological properties. *Phys. Rev.* B **93**, 205442 (2016).

[25] Luo, X., Sullivan, M. B. & Queck, S. Y. First-principles investigations of the atomic, electronic, and thermoelectric properties of equilibrium and strained Bi_2Se_3 and Bi_2Te_3 including van der Waals interactions. *Phys. Rev.* B **86**, 184111 (2012).

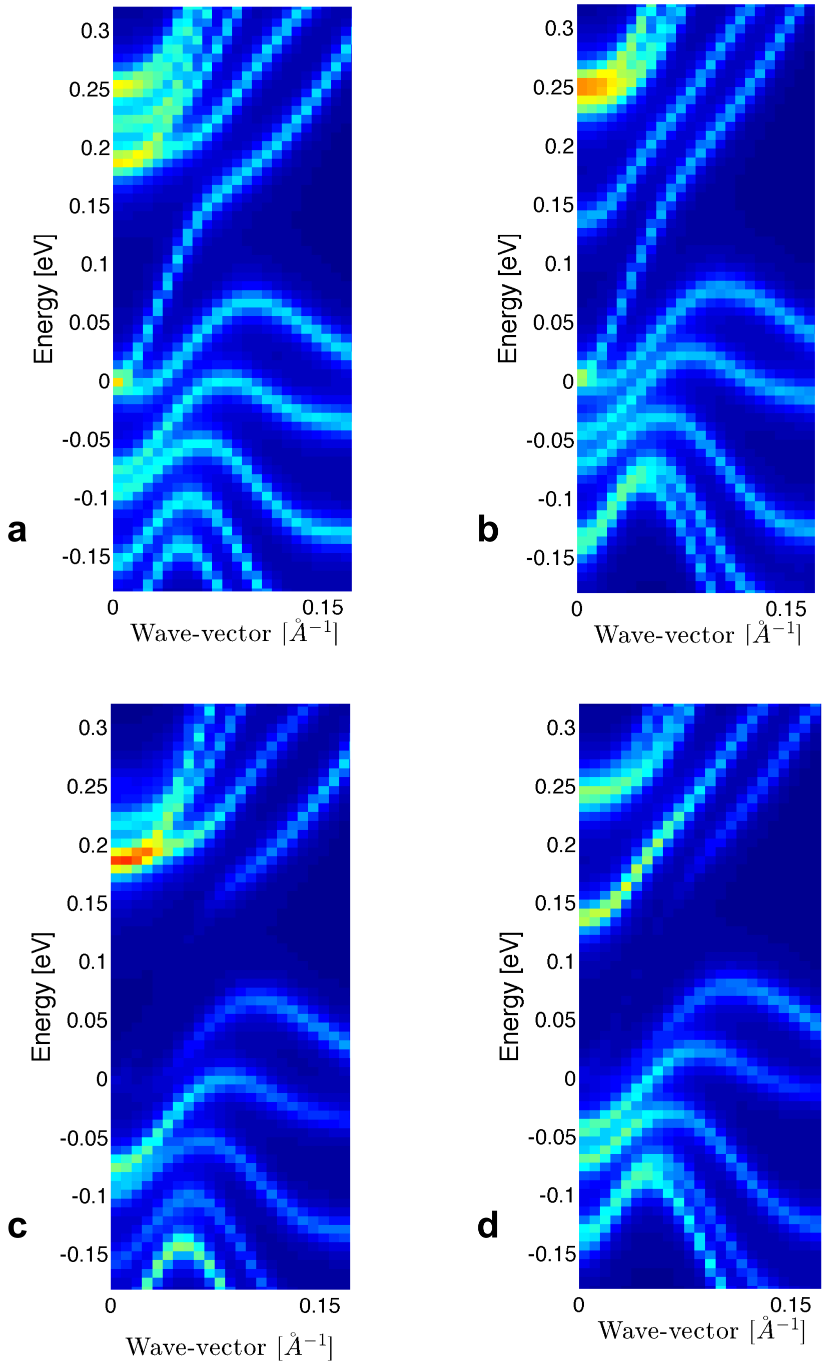

Supplementary Figure 1 Electronic properties of the stacking fault. Electronic band structure near the Γ-point of a 6 quintuplet Bi_2Te_3 system without a stacking fault (**a**) and with a stacking fault (**b**). Panels **c** and **d** show the same band structures where the weights of states contributing to the spectral function are obtained by projecting the wave functions on atomic sites situated in one of the middle quintuplets.

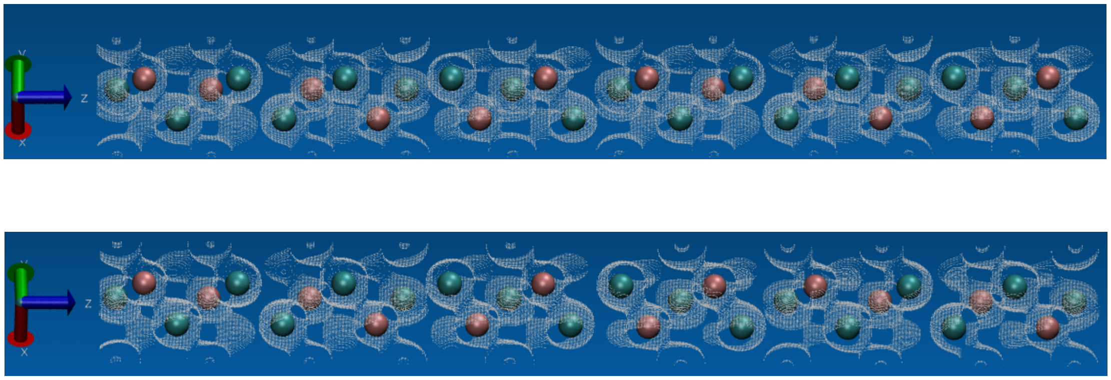

a

b

Supplementary Figure 2 Stacking fault effect on the charge density. Charge density plot for a relaxed 6 quintuplet system in Bi_2Te_3 without a stacking fault (**a**) and with a stacking fault in the middle (**b**). The quintuplet layers are normal to the z-axis.
